# Human Salmonellosis: A Continuous Global Threat in the Farm-to-Fork Food Safety Continuum

**DOI:** 10.3390/foods12091756

**Published:** 2023-04-23

**Authors:** Addisu D. Teklemariam, Rashad R. Al-Hindi, Raed S. Albiheyri, Mona G. Alharbi, Mashail A. Alghamdi, Amani A. R. Filimban, Abdullah S. Al Mutiri, Abdullah M. Al-Alyani, Mazen S. Alseghayer, Abdulaziz M. Almaneea, Abdulgader H. Albar, Mohsen A. Khormi, Arun K. Bhunia

**Affiliations:** 1 Department of Biological Sciences, Faculty of Science, King Abdulaziz University, Jeddah 21589, Saudi Arabia; ateklemariam@stu.kau.edu.sa (A.D.T.); ralbiheyri@kau.edu.sa (R.S.A.); mgalharbi@kau.edu.sa (M.G.A.); mamalgamdi2@kau.edu.sa (M.A.A.); afilimban@kau.edu.sa (A.A.R.F.); aalbar0030@stu.kau.edu.sa (A.H.A.); 2 Centre of Excellence in Bionanoscience Research, King Abdulaziz University, Jeddah 21589, Saudi Arabia; 3 Laboratory Department, Saudi Food and Drug Authority, Riyadh 12843, Saudi Arabia; asmutiri@sfda.gov.sa; 4 Laboratory Department, Saudi Food and Drug Authority, Jeddah 22311, Saudi Arabia; amalyani@sfda.gov.sa; 5 Monitoring and Risk Assessment Department, Saudi Food and Drug Authority, Riyadh 13513, Saudi Arabia; 6 Department of Microbiology and Medical Parasitology, Faculty of Medicine, Jeddah University, Jeddah 23218, Saudi Arabia; 7 Department of Biological Sciences, Faculty of Sciences, Jazan University, Jazan 82817, Saudi Arabia; makhormi@jazanu.edu.sa; 8 Molecular Food Microbiology Laboratory, Department of Food Science, Purdue University, West Lafayette, IN 47907, USA; 9 Purdue Institute of Inflammation, Immunology, and Infectious Disease, Purdue University, West Lafayette, IN 47907, USA; 10 Purdue University Interdisciplinary Life Science Program (PULSe), West Lafayette, IN 47907, USA; 11 Department of Comparative Pathobiology, Purdue University, West Lafayette, IN 47907, USA

**Keywords:** antibiotics resistance, farm-to-fork, foodborne, food safety, non-typhoidal *Salmonella*, outbreak, pathogenesis, *Salmonella*

## Abstract

*Salmonella* is one of the most common zoonotic foodborne pathogens and a worldwide public health threat. *Salmonella enterica* is the most pathogenic among *Salmonella* species, comprising over 2500 serovars. It causes typhoid fever and gastroenteritis, and the serovars responsible for the later disease are known as non-typhoidal *Salmonella* (NTS). *Salmonella* transmission to humans happens along the farm-to-fork continuum via contaminated animal- and plant-derived foods, including poultry, eggs, fish, pork, beef, vegetables, fruits, nuts, and flour. Several virulence factors have been recognized to play a vital role in attaching, invading, and evading the host defense system. These factors include capsule, adhesion proteins, flagella, plasmids, and type III secretion systems that are encoded on the *Salmonella* pathogenicity islands. The increased global prevalence of NTS serovars in recent years indicates that the control approaches centered on alleviating the food animals’ contamination along the food chain have been unsuccessful. Moreover, the emergence of antibiotic-resistant *Salmonella* variants suggests a potential food safety crisis. This review summarizes the current state of the knowledge on the nomenclature, microbiological features, virulence factors, and the mechanism of antimicrobial resistance of *Salmonella*. Furthermore, it provides insights into the pathogenesis and epidemiology of *Salmonella* infections. The recent outbreaks of salmonellosis reported in different clinical settings and geographical regions, including Africa, the Middle East and North Africa, Latin America, Europe, and the USA in the farm-to-fork continuum, are also highlighted.

## 1. Introduction

In 1884, Theobald Smith isolated *Hog-cholerabacillus* from pigs’ intestines infected with swine fever and named it *Salmonella* after his mentor, Daniel Elmer Salmon, an American veterinary pathologist. *Salmonella* is one of the common bacterial enteropathogens responsible for sporadic illness or widespread gastrointestinal disease [1,2,3,4]. Up to 80% of human *Salmonella* infections are not associated with a known outbreak; instead, they are considered a sporadic illness, and some are not diagnosed [5]. The World Health Organization estimated 153 million non-typhoidal *Salmonella* (NTS) infections worldwide in 2010, of which 56,969 were lethal and nearly half were foodborne [6]. A total of 926 salmonellosis foodborne outbreaks were reported across 23 European countries in 2019, resulting in 9169 cases, 1915 hospitalizations, and 7 deaths. Salmonellae were responsible for 17.9% (one in six) of all foodborne outbreaks in 2019 [7]. *Salmonella* is considered the most common foodborne organism in imported foods from Africa to the European Union. A large proportion (72.4%) of the foodborne salmonellosis outbreaks were caused by *Salmonella enterica* serovar Enteritidis [8]. The two most common serovars in the United States are *Salmonella enterica* serovar Typhimurium (*S*. Typhimurium) and serovar Enteritidis (*S*. Enteritidis), and these were responsible for 41.5% of the total outbreaks. These two serovars account for nearly 60% of all *Salmonella* outbreaks worldwide [9] and 91% of African cases [10].

Antimicrobial resistance in *Salmonella* is a significant concern for public health safety [11,12]. This pathogen must receive more attention, particularly its presence in the food/feed supply chain [13]. Food animals are often reservoir hosts for *Salmonella*; thus, it is difficult to eradicate this organism from them [14,15].

Outbreak studies have recognized food commodities, including fresh produce, vegetables, cantaloupes, cereals, alfalfa sprouts, fruit/fruit pulp, pistachios, poultry meat, turkeys, tuna, ground beef, pork, dried/shredded coconut, tomatoes, and mangoes, as sources of foodborne *Salmonella*-related outbreaks in the past decade [16,17]. The magnitude of infection that has been provoked due to antibiotic-resistant variants is recurrently implied as the causative organisms in these outbreaks, resulting in a higher level of systemic diseases, treatment failures, and a surge in hospitalizations [18,19].

This review provides an updated summary of the nomenclature, taxonomy, microbial properties, pathogenesis, transmission, and antimicrobial resistance of human salmonellosis. The recent outbreaks of human salmonellosis reported in different clinical and geographical settings, including the farm-to-table food supply continuum, are also presented.

## 2. Materials and Methods

Medline (PubMed), Web of Science, Embase, and Google Scholar were searched for relevant articles. The search string that enabled us to locate most studies was *Salmonella* AND (Africa OR Asia OR Europe OR Central America OR South America OR USA OR Middle East. Additional searches involved using the main medical subject headings (MeSH) terms with Boolean operators and other words that included epidemiology, transmission, virulence, pathogenesis, clinical manifestations, foodborne, and drug resistance. We performed the last search on 21 January 2023. The full texts or abstracts of all articles were screened for eligibility. Duplicates or research articles that did not meet the eligibility criteria were excluded. Studies that were non-English (language), nonbacterial, non-salmonella (organism), or nonhuman (host) were also omitted. 

## 3. Classification and Nomenclature

The nomenclature and naming of *Salmonella* are relatively complex and are still evolving. Based on variations in the sequence of the 16S rRNA gene, the *Salmonella genus* is classified into two species, *Salmonella bongori* and *Salmonella enterica* (type species). *S. enterica* can be categorized into six subspecies depending on their biochemical properties and genetic relationship [20,21,22]. The subspecies are designated with Roman numerals, as follows: I, *S. enterica* subsp. *enterica*; II, *S. enterica* subsp. *salamae*; IIIa, *S. enterica* subsp. *arizonae*; IIIb, *S. enterica* subsp. *diarizonae*; IV, *S. enterica* subsp. *houtenae*; VI, *S. enterica* subsp. *indica*. *Salmonella enterica* subsp. *enterica* (I) is the predominant subspecies in mammals, accounting for around 99% of human salmonellosis and *Salmonella* infections in warm-blooded animals. On the contrary, *S. bongori* and the other five subspecies are predominant in cold-blooded animals and the environment [23].

**Figure 1:**
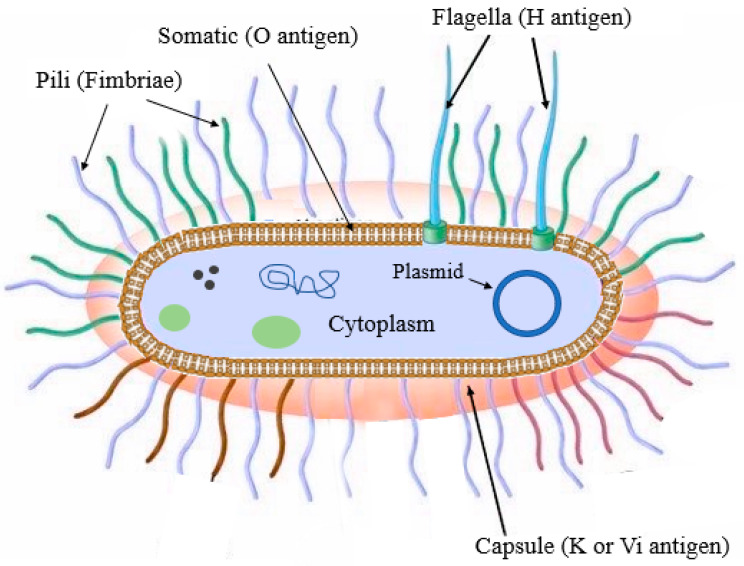
Schematic illustration of the structure of *Salmonella* (adopted from https://www.cram.com/flashcards/enterobacteriaceae-5178261 and modified using Biorendor software. (accessed on 11 October 2022)).

Besides phylogenetic classification, Kauffman and White developed a system of identifying *Salmonella* serotypes using three chief antigenic determining factors: flagellar (H), capsule (K), and somatic (O) antigens [23] (Figure 1). The O antigen is the heat-stable form of lipopolysaccharides found on the outer membrane. Some salmonellae serotype express one or more O antigen types [24]. H antigens represented by bacterial flagella stimulate the host’s immune response. Most *Salmonella* serovars contain two definite genomic regions that encode flagella synthesis. They have the unique capability to express only one of these proteins at a time, which is why they are called diphasic (phase I and II) bacteria. Phase I H antigens account for the immunological character of a serotype, while phase II antigens are common to many serotypes [25]. K antigens are polysaccharides that are sensitive to heat and attach to the surface of bacterial capsules. It is the least abundant antigen in all serotypes [26]. A particular subtype of the K-antigen called the virulence (Vi) antigen is detected only in three pathogenic serotypes (not all strains), namely Dublin, Typhi, and Paratyphi C [27].

The *Salmonella* serotypes can be identified by analyzing all the antigenic properties of the bacterium. An agglutination test using antibodies unique to the O antigens can be performed to classify the organism into six serogroups, namely A, B, C1, C2, D, and E. This approach offers crucial evidence for epidemiological study and enables genus and/or species-level identification [28]. Thus far, more than 2500 serovars have been identified; more than half of these serotypes are part of *S. enterica* subsp enterica, which is responsible for most human salmonellosis [29]. The taxonomy and nomenclature of *Salmonella* should follow a standard format (i.e., *Salmonella enterica* subsp *enterica* serovar Typhimurium, which is abbreviated as *S*. Typhimurium) while describing them in the literature [30]. 

More recently, Chattaway et al. [31] proposed a taxonomic classification based on whole genome sequence data to define a two-tier subtyping approach: multilocus sequence typing (MLST) followed by antigen prediction. Using this approach, the researchers classified 46,000 isolates of *Salmonella enterica* subspecies *enterica* that showed a 99.96% match to a serovar organized by the Kauffmann and White scheme. 

## 4. Pathogenesis

**Figure 2:**
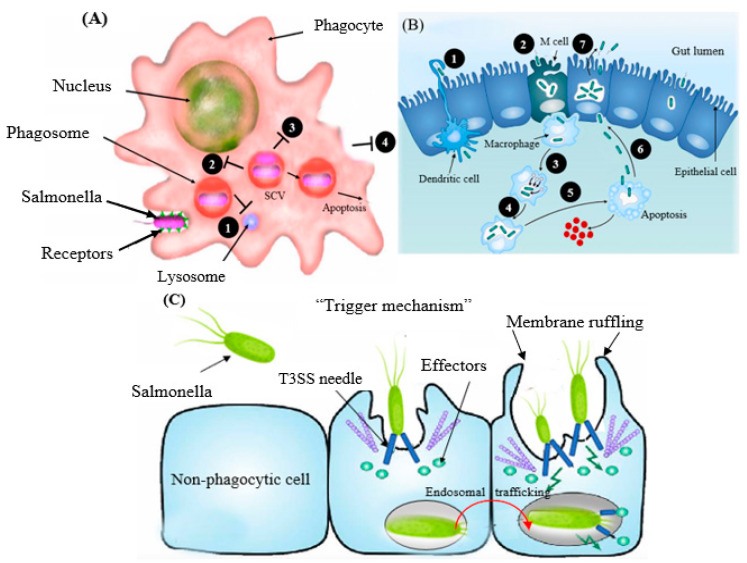
Schematic demonstration of (**A**) resistance mechanism of *Salmonella* to phagocytic destruction: 1. prevention of phagolysosome fusion, 2. resistance to oxidative and nitrosative stress, 3. resistance to antimicrobial peptides, 4. inhibition of macrophage-mediated inflammation). (**B**) Mechanisms of internalization (course of infection) of *Salmonella*. Once *Salmonella* reaches the lumen of the gut, they can pass the epithelial layer either by (1) a passive uptake expedited by dendritic cells; (2) invasion via the M cells; (3 and 4) bacterial replication and survival; (5) secretion of cytokines, inflammation, and/or apoptosis; (6 and 7) basolateral invasion to reseed back into the lumen of the gut, which leads to shedding of the agent via feces. (**C**) Colonization of non-phagocytic host cells (fibroblast, endothelial, and epithelial cells) by a trigger mechanism. SCV, *Salmonella*-containing vacuole.

The severity of human salmonellosis depends on the causative serotypes and the immune health of the patient or the presence of comorbid diseases in the infected individuals. The aging population, children under 5 years, and immunodeficient individuals are more vulnerable to *Salmonella* infections. *Salmonella* showed an unusual characteristic in the course of its colonization of non-phagocytic cells [32], whereby it triggers phagocytosis (referred to as trigger mechanism) to be able to enter the host cell (Figure 2A). These virulence factors are situated in *Salmonella* pathogenicity islands (SPIs), the cluster of genes positioned at the large region of the chromosomal DNA and standing for the structural domains that participate in the invasion process [33]. The bacterium tends to penetrate the intestinal wall epithelial cells after entering the digestive system via contaminated water or food. The transportation of *Salmonella* via the barrier of the intestine arises mainly through the specialized microfold (M) cells (Figure 2B) positioned in the lymphoid tissue, also named Peyer’s patches [34] or by active penetration of nonphagocytic cells, via the so-called “trigger” process [35] (Figure 2C). The type III secretion system (T3SS) is the cell surface multi-protein channel, which allows the bacterium to deliver its effector molecules (payload) into the host cytosol [36]. The effectors then activate the signal transduction system and induce the rearrangement of the host actin cytoskeleton, leading to the outward projection of the membrane of the epithelial cell to internalize the bacterium. The structure of the projected membrane resembles the process of phagocytosis, which is conducted by normal phagocytic cells [37] (Figure 2C).

The intracellular persistence of *Salmonella* is vital for pathogenesis, which may vary among hypervirulent to low-virulence strains [38,39]. After the engulfment, the bacterium is encased in a host-derived sheath of a membrane known as a vacuole (also named a *Salmonella*-containing vacuole, SCV) (Figure 2A). The host cell then triggers the fusion of the phagosome with lysosomes and the production of enzymes and reactive oxygen species to destroy the captured bacteria [40]. The bacterium injects effector proteins directly into the vacuole using the T3SS, resulting in the compartment’s structural modification. The modified vacuole structure stops the phagolysosomal fusion, which protects the organism in the intracellular niche to promote safe replication (Figure 2A). The ability of this organism to evade macrophages enables the bacterium to be transported in the reticuloendothelial system (RES) [41,42].

### 4.1. Salmonella Virulence Factors

#### 4.1.1. Pathogenicity Islands

There have been 24 *Salmonella* pathogenicity islands (SPIs) identified thus far. These horizontally obtained loci encode genes assisting many virulence factors, including (i) secretion systems, flagella, fimbriae, and capsule synthesis, (ii) colonization of the host cell, (iii) host evasion mechanisms and survival, and (iv) seroconversion [43]. Among the SPIs, approximately 21 SPIs are typically found in *S. enterica*, encoding genes required for pathogenesis, survival inside the host, and maintenance of housekeeping genes [44,45]. The genetic and phenotypic characteristics of *Salmonella* pathogenicity islands 1 (SPI-1) and 2 (SPI-2) have been well-researched. SPI-1 is commonly found in all species and subspecies [46]. T3SS encoded by SPI-1 is involved in the export of effector proteins for host cell colonization. Contrary to this, SPI-2 is present only in *S*. *enterica*, while SPI-22 is found only in *S. bongori* [46]. The SPI-2 gene encodes an additional T3SS critical for survival at the intracellular niche and for preventing SCV acidification. 

Several SPIs have been associated with *S. enterica* subsp *enterica* serovars, and some have been reported to confer fitness advantages on specific serovars. Although SPI-7 encodes the Vi capsule, it was traditionally thought to appear exclusively in strains of *S*. Typhi [47]; however, it has also been found in NTS serovar Dublin [48,49]. These two serovars have been associated with invasive diseases in humans. Some serovars carry typhoid toxin genes on SPI-11, even though SPI-11 is common among *S. enterica* subsp *enterica* [50]. Researchers have reported that carriers of the typhoid toxin gene *cdtB* (cytolethal distending toxin) are more likely to develop invasive diseases [51]. It has also been proposed that certain SPIs can help colonize a specific host. T6SS located in SPI-19 was shown to be responsible for *S*. Gallinarum and *S*. Typhimurium colonization and spread to deeper tissues in chickens [52].

#### 4.1.2. Toxins

Bacterial toxins that are released extracellularly and act on the target host cells are exotoxins. The toxin produced by serotype Typhi, designated the “typhoid toxin”, is responsible for the pathogenesis and typhoid symptoms [53]. Typhoid toxin is also called *Salmonella cdt*, and at least 41 NTS serovars have been reported to harbor this genotoxin [50,54]. However, it has not been found in serovars Enteritidis, Typhimurium, or Newport. Scientists have discovered that the *S*. Typhi toxin helps to fine-tune the host’s defense against infection by marking specific target cells, including immune and brain cells [55]. 

*S*. Typhimurium DT-104 and other NTS strains also encode an exotoxin called ArtAB toxin (ADP-ribosylating toxin) [51]. The ArtA and ArtB subunits form the active and pentameric binding subunits, respectively [56]. These two subunits share homology with pertussis toxin, produced by *Bordetella pertussis* [56]. 

Several reports indicate that various NTS serovars also secrete trypsin-sensitive, heat-labile cytotoxins. In some cases, the cytotoxic effect is related to the outer membrane. It has been reported that some serovars are capable of producing cytotoxins, including Nchanga, Enteritidis, Saintpaul, Braenderup, Indiana, Choleraesuis, Virchow, and Typhimurium [57]. 

#### 4.1.3. Flagella

Flagella are a locomotory cellular appendage in most NTS [43,58], except for serovar Gallinarum biovars Pullorum and Gallinarum [59]. Research findings indicate the involvement of 50 or more genes in synthesizing flagella assembly and maintenance [60]. Nevertheless, the building block, flagellin, is encoded individually by *fliC, fljB,* and *flpA* genes for phases 1, 2, and 3, respectively [61]. Most NTS serovars express 5–10 peritrichous flagella [61]. Phase variation is one of the crucial characteristics of different *Salmonella* serovars, which is a genetic reshuffle (reversible) to switch between the expression of *fliC* and *fljB*. This method is developed by several bacterial pathogens [62,63].

Flagella help *Salmonella* to move toward the host epithelial layers and are also a potent inducer of the innate immune response in the host [64]. Additionally, it has been demonstrated that flagella enable the organism to move toward the tetrathionate and nitrate generated by the host microbiota, which is utilized as alternative terminal electron acceptors for survival inside the host gut [65,66].

#### 4.1.4. Fimbriae (Pili)

A thin bacterial cell surface appendage called fimbriae aids the organism in adhering and attaching to the host cell. This structure is produced by several gram-positive and gram-negative bacteria [67]. Genomic and phenotypic investigations have identified the 39 distinct fimbrial operons in *Salmonella.* Of all operons, *agf* is found in both *S. bongori* and *S. enterica* and encodes the nucleator-dependent curli fimbriae, which are aggregative thin structures that help in firm attachment and colonization processes [68]. The type IV fimbriae are encoded by *pil* and *bfp* operons; the former operon is present on SPI-7. Hence, it is limited to serovar Paratyphi C, Typhi, and Dublin [48]. The other 36 fimbrial operons encode chaperone-usher-dependent fimbrial (CUDF) pathways [68]. Twenty-seven fimbrial operons have been detected in NTS [68]. The *fim* operon is the only CUDF operon found in all *S. enterica* isolates [68]. Many fimbrial genes are not expressed in in vitro environments but are detected during in vivo infection [69].

#### 4.1.5. Protein Secretion System

**Figure 3:**
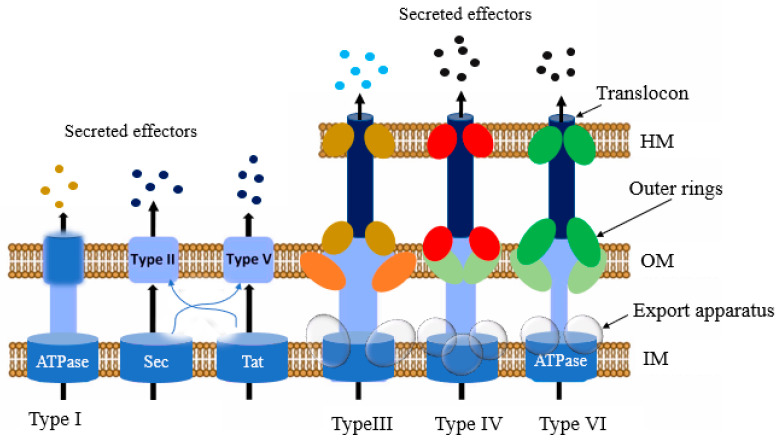
Schematic elucidation of the different types of secretion systems that participate in *Salmonella* infection, namely I (T1SS), III (T3SS), IV (T4SS), and VI (T6SS). HM, host cell membrane; IM, bacterial inner membrane; OM, outer membrane.

Presently, four protein secretion systems have been identified in *Salmonella*, including the type I (T1SS), III (T3SS), IV (T4SS), and VI (T6SS) secretion systems (Figure 3). According to reports, these systems play a pivotal role in host infection [70]. They are mainly used to transport and translocate the bacterial effector proteins and to transport some (T3SS and T4SS) directly into the target cell cytoplasm, and to modulate signaling cascades of the host cell [71,72,73]. In the host cytosol, the effector proteins can change normal cellular activities, such as membrane transport, cytoskeleton structure, cytokine expression, and signal transduction [74]. The secretion apparatus contains numerous components comprising over 20 different types of proteins [75]. Some proteins are homologous to the proteins involved in flagellar assembly, indicating an evolutionary relationship between the two [76].

Among all the secretion systems, T3SSs are the most advanced and well-studied secretion system in bacteria [75,77,78]. *Salmonella* outcompetes the gut commensal bacteria with the help of T6SS. T1SS and T3SS-1 trigger this organism’s adherence to the intestine’s epithelial layer. After that, they facilitate invasion of M cells or epithelial cells by releasing effectors via T3SS-1 and T6SS. Once internalized, T3SS-2, T4SS, and T6SS promote the replication and survival of *Salmonella* in SCVs [70].

#### 4.1.6. Biofilm Production

Besides the planktonic phase, *Salmonella* sometimes exists as a sessile or multicellular form (also known as biofilm) that enable the agent to firmly adhere to biotic and/or abiotic surfaces [79,80,81]. Biofilm formation has been linked to *Salmonella* persistence on food surfaces, plants, and other fresh produce, and biofilm protects the pathogen during food processing [82]. Forming biofilm contributes to the dissemination of *Salmonella*, since microbes in the biofilm are protected from chemical, mechanical, and physical stressors [79]. It also contributes to the virulence of *Salmonella*, since bacteria in the biofilm are not vulnerable to the host’s immune attack and antimicrobials, leading to the long-term survival of the agent, which ultimately results in chronic infection and carrier state [83,84]. The biofilm formed by *Salmonella* is mainly made up of O antigen, curli (amyloid fimbriae), biofilm-associated protein (Bap), cellulose, and extracellular DNA [79,80,85]. Curlin subunit gene D (CsgD), regulated by transcriptional factors, bis-3′-5′-cyclic dimeric guanosine monophosphate (c-di-GMP), and sRNAs, regulates biofilm formation by *Salmonella* [86]. The biofilm-forming capacity may vary among serovars [87] but largely depends on various extrinsic factors, including temperature, source, and contact surface [88,89]. In addition, biofilm formation may vary based on their carrier state, i.e., chronic carriage versus sensitive isolates of *Salmonella* [90].

## 5. Clinical Manifestations

*Salmonella* causes two forms of the disease in humans: typhoid fever and gastroenteritis. The latter is also known as non-typhoidal salmonellosis [4]. Human salmonellosis has four clinical manifestations: gastroenteritis, bacteremia, enteric fever, extraintestinal complications, and chronic carrier state [91].

### 5.1. Enteric Fever

*Salmonella enterica* serovar Typhi causes typhoid fever, while *S.* Paratyphi A, B, and C cause paratyphoid fever. As the clinical signs of typhoid fever are different from that of paratyphoid fever, the term “enteric fever” (EF) is used to describe both fevers, and both of these agents are called typhoid *Salmonella* [92,93]. Humans are the only reservoir for both *S.* Typhi and *S.* Paratyphi, collectively called typhoid salmonellae. Humans acquire the disease via ingesting water or food contaminated with the biowaste of infected individuals. EF onset needs a one or more weeks incubation period, with initial symptoms, such as abdominal pain, headache, and constipation (or diarrhea), accompanied by the onset of fever [94]. Diarrhea is usually seen in children, whereas constipation is more often observed in immunodeficient patients [95]. In the course of the illness, enteric fever exhibits two phases, with an initial low-grade (>37.5 °C to 38.2 °C) gradually reaching a high-grade (>38.2 °C to 41.5 °C) fever in the second week. In untreated patients, the fever may linger for a month or more [96]. Infected individuals may also show bradycardia, myalgia, splenomegaly (enlarged spleen), hepatomegaly (enlarged liver), and rose spots on their abdomen and chest [97]. Nearly 15% of diseased individuals in an endemic area develop gastrointestinal tract (GIT) complications, including hepatitis, cholecystitis, and pancreatitis. Bleeding is one of the GIT complications that follow the puncture of Peyer’s patches, lymphatic nodules situated at the ileum termini, leading to dysentery. Furthermore, the capability of typhoid salmonellae to persist and survive in the reticuloendothelial system causes a relapse in nearly 10% of the infected individuals [98].

### 5.2. Gastroenteritis

**Figure 4:**
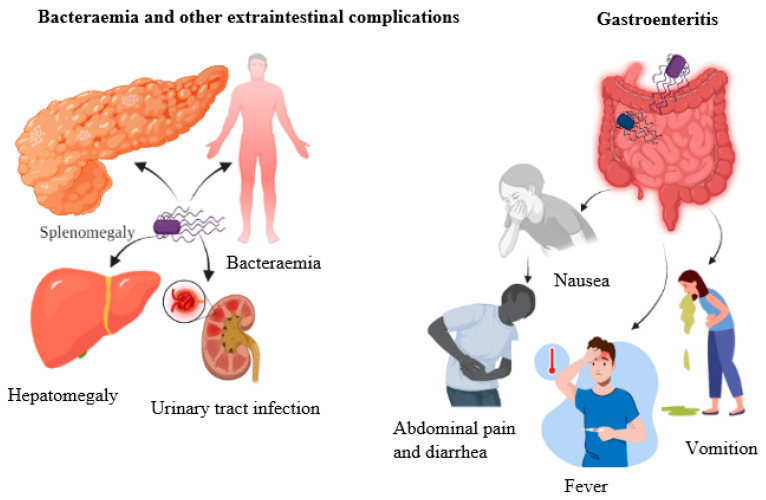
Clinical manifestation of human salmonellosis. The first clinical presentation is gastroenteritis complemented by headache, nausea, vomiting, abdominal pain, non-bloody diarrhea, and myalgia. The second clinical manifestation is bacteremia, other extraintestinal complications, and splenomegaly, hepatomegaly, and urinary tract infections.

Salmonellae serovars other than *S*. Paratyphi and *S.* Typhi are called NTS and are present in animal reservoirs. Gastroenteritis, an inflammatory condition of the GIT, is a characteristic feature of NTS infection, which is complemented by other clinical signs, such as headache, nausea, vomiting, abdominal pain, non-bloody diarrhea, and myalgia. Splenomegaly and hepatomegaly are less frequently encountered in NTS-infected patients [99] (Figure 4). Compared to typhoid infections, NTS has an incubation period of 6–12 h, and the clinical signs are often self-limiting and linger for 10 or a few days [100]. GI complications of NTS infections include cholecystitis, appendicitis, and pancreatitis without causing terminal ileum perforation [99]. The severity of the disease and its symptoms would be higher in susceptible individuals, such as immunocompromised patients, aging populations, infants, and young children [101]. Invasive NTS (iNTS) is prevalent in malnourished and immunocompromised individuals and is widespread in sub-Saharan Africa, resulting in high mortality [10,102].

### 5.3. Bacteremia and Other Extraintestinal Complications

*Salmonella* bacteremia occurs when *Salmonella* invades the intestinal barrier and enters the bloodstream. Almost all serotypes of *Salmonella* can cause bacteremia; however, *S.* Cholearaesuis and *S.* Dublin are the two highly invasive serovars strongly linked with bacteremia [27,103]. In contrast to typhoid salmonellae infections, NTS infections are more likely to cause bacteremia. Based on genetic analysis, some serotypes, such as Enteritidis, Typhimurium, and Dublin, but not all serotypes, are suspected of possessing the *Salmonella* plasmid virulence gene (*spv*), which can cause non-typhoidal bacteremia [104]. While it is unclear how the presence of the gene increases NTS virulence, expression of the gene prolongs apoptotic cell death, which may permit the pathogen to survive in the host longer [105]. About 5% of patients who contract NTS develop bacteremia, and the lung is the most impacted organ in some cases. Urinary tract infections (UTIs), cellulitis, pneumonia, meningitis, and endocarditis are the other notable extraintestinal complications [106,107].

### 5.4. Carrier State and Transmission

**Figure 5:**
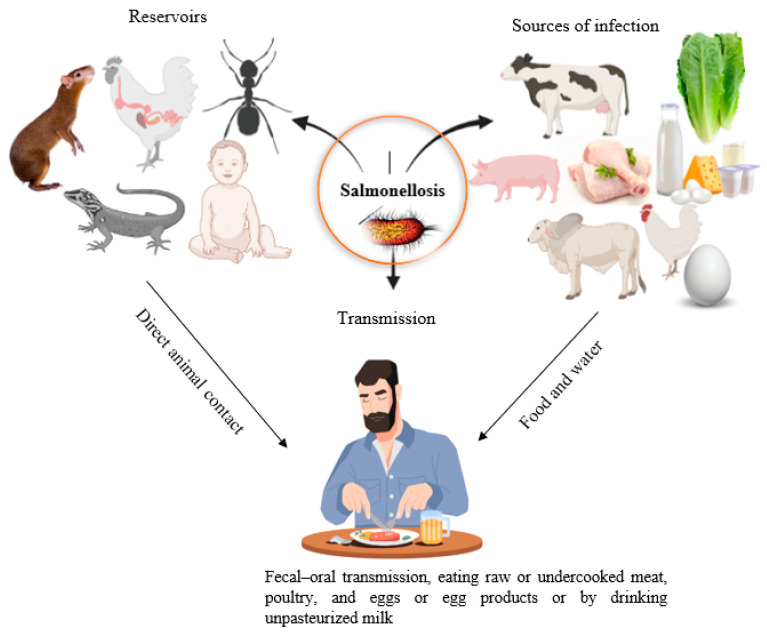
Diagram showing the primary reservoir, source of infection, and the transmission mechanism of human salmonellosis. Insects, reptiles, humans, and animals can serve as a reservoir for human salmonellosis. Uncooked, undercooked, and/or contaminated foods (poultry, beef, pork, egg, milk, milk products, vegetables, flour, nuts, etc.) are familiar sources of infection.

The *Salmonella* chronic carrier state is defined as when bacteria are shed in the stool for at least one year after the onset of the acute infection. Colonization in the gall bladder and secretion through bile is considered a prime source of fecal shedding [108]. In endemic regions, carriers of *S.* Paratyphi and *S.* Typhi are responsible for the spread of enteric fever, and the most common means of transmission is the fecal–oral route (drinking of water or ingestion of food contaminated by the feces of chronic carriers) [109] (Figure 5). Nearly 4% of enteric fever patients, including infants, the aging population, and women, may serve as chronic carriers [108]. On the other hand, a carrier state of NTS occurs in only 0.1% of patients, because animals are the primary source of NTS, rather than humans. 

*Salmonella* is a prototype foodborne pathogen whose transmission between animals, plants, and humans has been well-documented [110]. *Salmonella* can spread from one farm building and facility to another via pests, including flies, mice, rats, and cockroaches [111,112,113]. *Salmonella* can be carried in the intestinal tracts of rodents asymptomatically without causing any clinical symptoms and serve as vectors and reservoirs of the disease (Figure 5). It has also been documented that bacteria are transmitted from cattle to humans through flies, which serve as mechanical vectors [113]. Wildlife, including wild birds, is a pivotal reservoir for *Salmonella* infection [114]. Studies have shown that human or farm workers’ movement among different farms increases the risk of disease in chickens, hens, and pigs [115,116].

## 6. Epidemiology

Among *Salmonella* infections, NTS infections are the most common cause of self-limiting illness. Enteric fever caused by typhoid *Salmonella* has a high mortality and morbidity rate and occurs more frequently in developing nations [117,118]. 

### 6.1. Epidemiology of Enteric Fever

Enteric fever (EF) is endemic in different regions of Asian and African nations and countries in Europe, Central and South America, and the Middle East. EF is rare in the U.S. and some other European countries, with fewer than 10 cases of salmonellosis per 100,000 people each year. Most reported cases in these countries are linked to international travel. Travelers returning from India, Africa, or Pakistan are often the source of this disease [119,120,121]. The rise in cases of *S.* Paratyphi infection raises concerns about the efficacy of current vaccines for typhoid fever and suggests the need for a more comprehensive study.

Enteric fever is prevalent in several Asian nations, such as Indonesia, India, Vietnam, China, and Pakistan, with yearly incidence rates surpassing 100 cases per 100,000 people [122]. Since the data collected by EF are from significant outbreaks, the global incidence of EF reports is more of an estimate. Because of the shortage of diagnostic facilities and effective surveillance technologies in many developing nations, predominantly in sub-Saharan Africa, the prevalence of EF is poorly characterized [123].

### 6.2. Epidemiology of Non-Typhoid Salmonella Infections

Despite advances in sanitation and hygiene, the number of NTS infections remains high, posing a problem in developed and developing nations [2,124,125]. Invasive NTS capable of spreading to extraintestinal sites is prevalent in developing countries, particularly in sub-Saharan Africa, with high incidence rates in children under three and HIV-positive individuals [124,126]. In Asia, the invasive illness produced by NTS is less common [127].

Inadequate cooking of foodstuffs, improper storage, and direct contact with raw ingredients are all considered significant causes of *Salmonella* outbreaks. Animal commodities, such as milk, poultry, eggs, and other foods, such as peanut butter and chocolate, are frequently linked to epidemics [128]. Most recently, onion has been implicated in salmonellosis outbreaks in the U.S. [129].

Animals are considered the primary reservoir of NTS [130]. Consumption of water or food contaminated with the excrement of infected animals, direct contact with infected animals, or ingestion of infected food animals can cause NTS infection in humans. The global incidence rate of NTS infection is high, as the strains may exist naturally and in wild and domestic animals, such as dogs, cats, amphibians, rodents, and reptiles [131]. Widespread distribution of food animals, wildlife, and various commodities are primary factors in salmonellae spread in the farm-to-fork food supply continuum. 

### 6.3. Outbreaks of Salmonellosis in Humans

When two or more individuals are afflicted with the same sickness from the same source of contaminated drink or food, such a scenario is known as a foodborne outbreak. Likewise, when two or more individuals suffer from the same disease from animal or animal products and associated environments, the event is classified as a zoonotic outbreak [132,133]. A brief overview of outbreaks of salmonellosis in humans on different continents is provided below.

#### 6.3.1. Africa

In Africa, NTS infections appear to be endemic, and are one of the major causes of bacteremia, mostly in children, with 4100 deaths per year [125]. The prevalence rate is higher in areas where malnutrition, malaria, and HIV are prevalent. Nearly 85.8% of global iNTS deaths have been reported in sub-Saharan Africa [2]. About 14.3 million typhoid and paratyphoid fever cases in 2017 resulted in 135,900 deaths, 15.8% of which were in sub-Saharan Africa [134].

*Salmonella* Typhi is the leading cause of bloodstream infections in eastern and southern Africa, with reports of multiple outbreaks since 2012 [135]. Malawi has a very high incidence of 444 cases per 100,000 persons per year [92]. The primary infection source of people’s exposure to *S*. Typhi is uncertain [136]. In Africa, iNTS is mainly associated with HIV patients (both adults and young children), malaria infection, and malnutrition [137]. Two *Salmonella* serotypes, Enteritidis and Typhimurium, have been reported to be the most common causes of iNTS in South Africa, Malawi, Mozambique, Kenya, and Mali, with *S*. Typhimurium Sequence Type (ST) 313 (ST313) and S. Enteritidis ST11 being the most frequently reported serovars [138]. In South Africa (2020 and 2021), although the total number of enteric fever cases across the country was similar to previous years (83 patients in 2020 and 110 patients in 2021), there was a relative upsurge in the number of cases reported from the northwest provinces and Western Cape [139]. In Nigeria, out of 372 humans screened, 77 (20.7%) were positive for enteric fever, 38 (20.4%) were isolated from non-poultry workers, and 39 (21.0%) were isolated from poultry staff in the three senatorial districts [140]. A recent study on 16,236 children from Kenya indicated that 1.3% of bloodstream infections was caused by *Salmonella* Typhimurium and Enteritidis, while *Salmonella* Typhi caused 1.4% of disease. *Salmonella* Enteritidis and *S*. Typhimurium were not significantly associated with rearing domestic animals. However, rearing chicken was linked to a high prevalence of *S.* Typhi (2.1%) infection. The rate of children infected with *Salmonella* Typhimurium and Enteritidis was significantly higher in households that used water pots as water storage containers compared to using water directly from the tap (0.6%) [141]. 

An extensive drug-resistant (XDR) strain of *Salmonella* Typhimurium was reported to cause millions of bloodstream infections per year in sub-Saharan Africa, including in the Democratic Republic of Congo (DRC) [142]. A recent study conducted in Burkina Faso indicated that among the 106 *Salmonella* isolates (77 human stools; 14 sandwiches), O antigen-positive *Salmonella* was confirmed in 86% (91/106) of the samples, and serogroup O:4,5 was the most common serogroup detected (40%; 36/91). *Salmonella* Enteritidis and Typhimurium represented 5.5% (5/91) and 3.3% (3/91), respectively, and were identified only from clinical isolates. Furthermore, 14 serotypes of *Salmonella* (12/91 human strains and 2/15 sandwich strains) were evocative of Kentucky and Bargny serotypes [143]. In Ethiopia (from 2010 to 2020), the pooled prevalence of *Salmonella* among human stools and animal-origin foods was 4.8% and 7.7%, respectively [144].

#### 6.3.2. Middle East and North Africa

Several reports indicate a worrisome rising trend of NTS cases in developing countries, including the Middle East and northern Africa (MENA) [145,146]. A systematic review and meta-analysis study conducted on the prevalence of enteric NTS in humans in the MENA countries indicated that there were 6356 *Salmonella*-positive cases associated with 252,831 human samples. The pooled *Salmonella* prevalence in MENA was estimated at 6.6%. The highest pooled prevalence of *Salmonella* reports were in Tunisia (10.2%), Morocco (17.9%), and Sudan (9.2%), while the lowest were in Oman (1.2%), Jordan (1.1%), and Palestine (1.2%) [147].

A recent study in Iran indicated that nearly 94% of *Salmonella* isolates were recovered from ≤5-year-old patients, and 99% were NTS. The author found extensive diversity among *Salmonella* isolates; serogroup D (46%) was predominant, and *Salmonella* Enteritidis (41%) was the most common serotype that showed the highest antimicrobial susceptibility rate (>96%). *S*. Newport from human specimens was isolated for the first time in Iran. Most isolates were sensitive to all antimicrobials tested, but 35% of isolates were not-typed (NT), which showed the highest resistance, with 48% being resistant to ≥1 antimicrobial tested [148]. 

Malaeb et al. [149] reviewed published data from Lebanon on *Salmonella* susceptibility/resistance patterns and its clinical complications. The estimated incidence was 13.34 cases per 100,000 individuals, and most cases occurred in the 20–39 age group with no significant gender variation. Poor and less developed districts of Lebanon had the highest number of cases, and the peak incidence was in summer [149]. 

A case-control study conducted in central Israel indicated that in 18 years (2001–2018), 34 cases of NTS were identified in the bloodstreams of infected patients. The median age was 59 years, with 20% of patients below 20 years of age [150].

*Salmonella* infection in Saudi Arabia is highly prevalent during the Hajj and Umrah seasons due to the gathering of many pilgrims [151]. A retrospective descriptive study conducted in King Khalid University Hospital (KKUH), Riyadh, Saudi Arabia, between May 2017 and December 2018, indicated 22 patients with invasive *Salmonella* infection. Fifteen (68%) were females, and seven (32%) were males. The range of ages was from 8 months to 74 years [152]. 

#### 6.3.3. Latin America

Typhoid is broadly accepted to be endemic in parts of Latin America; the region has a medium incidence of typhoid fever (53 per 100,000 people), corresponding to >273,000 cases annually [153]. Using cases reported to the National Public Health Surveillance System in Columbia between 2012 and 2015, typhoid salmonellae was found in 836 patients, with the majority (676/836; 80.1%) of reported cases originating from only 7 departments. They further characterized 402 *S*. Typhi isolates with available corresponding data recovered from various departments of Colombia through antimicrobial susceptibility testing and molecular subtyping. The majority (235/402; 58.5%) of these typhoid cases occurred in males aged between 10 and 29 years (218/402; 54.2%), with 3 deaths (0.74%). The overwhelming preponderance (339/402; 84.3%) of *S*. Typhi were susceptible to all tested antimicrobials. The organism showed the most resistance against ampicillin (30/402;7.5%), followed by nalidixic acid (23/402, 5.7%) [154]. 

In Brazil, serotyping of 3113 *Salmonella* isolates collected by the National Reference Laboratory for Enteric Diseases between 2011 and 2020 revealed 61 serogroups [155]. Calarga et al. [156] studied the prevalence of the antimicrobial-resistant phenotype in 789 NTS strains collected between 2000–2019 in São Paulo, Brazil. Among the non-susceptible isolates, 31.55, 14.06, and 13.18% were resistant to aminoglycosides, tetracycline, and β-lactams, respectively. Moreover, 68 and 11 isolates were MDR and extended-spectrum β-lactamase (ESBL) producers, respectively, whereas one isolate was colistin-resistant [156].

#### 6.3.4. USA

The Centers for Disease Control and Prevention (CDC), USA, estimates that approximately 1.35 million illnesses, 26,500 hospitalizations, and 420 deaths occur due to NTS infection each year in the U.S., resulting in an estimated $400 million in direct medical costs [157]. Between 2009 and 2011, antibiotic-resistant *Salmonella* strains that had developed resistance to 5 or more antibiotics caused over 66,000 illnesses in the U.S. [158]. According to CDC, antibiotic-resistant NTS infections are on the rise, approaching an estimated 10% for ciprofloxacin, 3% for ceftriaxone, and 1% for azithromycin [157]. Prolonged hospitalization and increased risk of bloodstream infections, treatment failure, and excess mortality have been associated with antimicrobial drug-resistant NTS infections [102]. 

In late 2022, a multi-country outbreak of *Salmonella* Typhimurium was reported in the USA and UK. The outbreak was associated with chocolate produced in Belgium and was distributed globally to over 113 countries and territories across all WHO regions. While 150 of 151 known cases have been reported in Europe, 1 case has been reported in the U.S. Additional cases are likely reported from other countries, given the broad distribution of the products during the Easter holiday [159].

#### 6.3.5. Europe

Salmonellosis remains the second most common zoonotic disease in humans in the European Union (EU). The incidence of human salmonellosis has decreased steadily in recent years. Nevertheless, in 2014, 88,175 confirmed human salmonellosis cases, causing 9830 hospitalizations and 65 fatalities, were reported across the EU. Among these, 16,000 cases of human salmonellosis were reported in Germany. As in previous years, *S.* Enteritidis was the predominant serovar (44.4% of all isolates), followed by *S.* Typhimurium (17.4%) and a monophasic *S*. Typhimurium variant (7.8%) [160].

After a considerable decrease in salmonellosis cases recorded from 2007 to 2014, the incidence was stable between 2015 and 2019. The number of cases in 2020 was significantly lower than in previous years, mainly due to the COVID-19 pandemic. All but two countries reported a decrease in the number of patients due to various factors, including people avoiding hospital and/or clinic visits for mild sickness for fear of the risk of exposure to COVID-19 in healthcare facilities, lower laboratory services because of the reallocation of resources to SARS-CoV-2, limited restaurant visits, frequent hand washing practices, and limited human movement and personal contacts due to travel restrictions [133]. 

Notification rates for human salmonellosis also differ between member states in the EU, including area coverage, quality of data, disease severity, surveillance systems, sampling and testing, the prevalence in the food-producing animal population, food and animal trade between member states, and the proportion of travel-associated cases [161].

In 2020, the majority (58%) of foodborne outbreaks were caused by *S.* Enteritidis, similar to previous years. The four most commonly encountered food vehicles in confirmed foodborne outbreaks associated with salmonellosis include ‘eggs and egg products’, ‘pig meat and products thereof’ and ‘bakery products”, as in previous years. Nearly 29 countries reported 53,674 cases, of which 53,169 were classified as confirmed. The number of notifications per 100,000 population was 14.2, considerably fewer than in 2019. Age-standardized notification rates did not differ substantially from crude rates. Of 35,715 cases with known outcomes, 61 were reported to have died, accounting for a case fatality of 0.17%. The highest prevalence was reported by the Czech Republic (98.4 cases per 100,000 population) and Slovakia (62.1), followed by Malta (34.2) and Hungary (30.3) [133]. Some of the recent outbreaks of human salmonellosis reported from different geographic regions associated with various foodstuffs are summarized in Table 1. 

## 7. Antimicrobial Resistance

Antimicrobial agents have been extensively used and misused for treating infectious diseases in animals and humans and as promoters of growth in livestock production [168,169]. Indiscriminate antibiotic use has led to the evolution of antimicrobial resistance (AMR) against antibiotics available to combat bacterial infections. AMR bacteria have emerged along the food chain, posing critical concerns to public health. Many studies have indicated the colonization, disease, and contamination of food animals and their products by one or more of these bacteria [169]. These include methicillin-resistant *Staphylococcus aureus* (MRSA), *Campylobacter* spp., and extended-spectrum beta-lactamase *Enterobacteriaceae*, such as *Salmonella*, *E. coli*, and *Shigella* [170].

Antimicrobial resistance in *Salmonella* has become an essential concern to public health worldwide [171]. The first case of *Salmonella* resistance to a single antibiotic, chloramphenicol, was reported in the early 1960s. Since then, nations, including the United Kingdom, United States, and Saudi Arabia, have seen an upsurge of antibiotic-resistant *Salmonella* isolates resistant to one or more antimicrobials [172]. Conventional first-line therapies for *Salmonella* infections include chloramphenicol, ampicillin, and trimethoprim-sulfamethoxazole [173]. Multi-drug resistant (MDR) salmonellae exhibit resistance to various drugs. A recent increase in MDR *Salmonella*, and resistance to clinically significant antimicrobials, such as third-generation cephalosporins and fluoroquinolones, pose substantial concerns.

The European Union report on antimicrobial resistance in human indicators and zoonotic bacteria from 2019 to 2020 indicates an increased resistance of salmonellae to sulfonamides, ampicillin, and tetracyclines at high levels (50–80%) [174]. However, the resistance to third-generation cephalosporins, namely ceftazidime and cefotaxime, was observed at very low levels of 0.5% and 0.8%, respectively. Higher MDR was noted in Italy and Belgium, with a 42.9% and 35% prevalence, respectively, from 2016 to 2020 (Table 2) [174].

### 7.1. Mechanisms of Antibiotic Resistance

A variety of hybrid plasmids (plasmids that contain an inserted piece of foreign DNA) are produced by *Salmonella* strains with the MDR phenotype. Plasmids carrying various resistance genes can confer resistance to conventional antibiotics, such as streptomycin, chloramphenicol, tetracycline, and ampicillin [175,176,177]. Several serotypes are also resistant to ciprofloxacin due to the mutations in the chromosomal-linked gene, *gyrA*, which also contributes to quinolone resistance [178]. Resistance to broad-spectrum cephalosporins is attributed to altered genes encoding extended-spectrum β-lactamases, which cleave β-lactam rings of cephamycins and cephalosporins [179,180].

**Figure 6:**
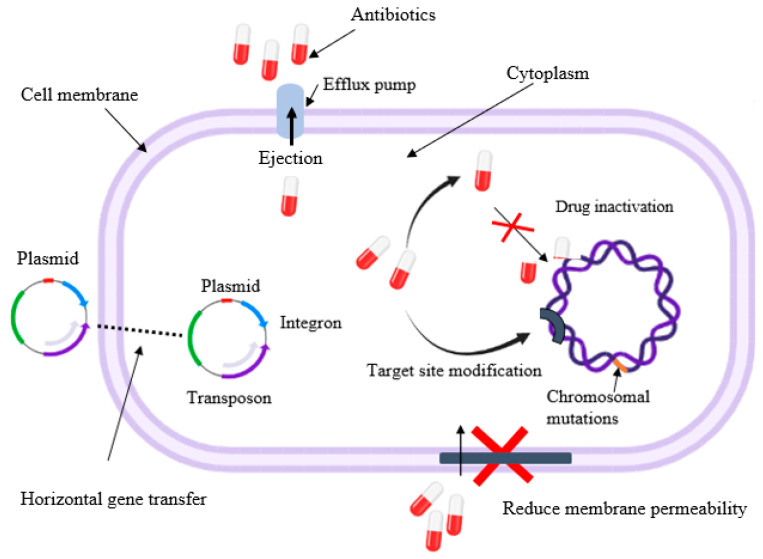
Schematic showing antibiotic resistance mechanisms in *Salmonella* spp.

Salmonellae use a variety of resistance mechanisms to combat antimicrobials, and drug inactivation is the most common (Figure 6) [177,181,182,183]. In this pathway, the functional unit of the antibiotic is inactivated or destroyed via chemical modification with the help of enzymes that hydrolyze phosphorylation, acetylation, and adenylation reactions [182]. Moreover, enzymes, such as chloramphenicol acetyltransferase and penicillinase, can acetylate the two hydroxyl groups of chloramphenicol, and ß-lactam rings of penicillin and cephalosporin, respectively [182].

Hindering the target site (cellular structure or enzymes) of antibiotics is the other mechanism of resistance that *Salmonella* serotypes can use. For example, quinolone resistance protein (Qnr), a plasmid-encoded protein, confers quinolone resistance by competing with topoisomerase IV and DNA gyrase for binding sites [182]. As a result, the antibiotic is less likely to bind to DNA gyrase, defending the bacteria from lethal effects [184]. Moreover, *Salmonella* can alter the receptor of the antibiotic in a way that prevents it from binding to it. Rifampicin resistance, for example, is caused by a single-step mutation that results in substitutions of amino acids in the *rpoB* gene.

Consequently, the affinity of the antibiotics for DNA polymerase could be lessened, permitting *rpoB* transcription to proceed [182]. It is also known that *Salmonella* resists drugs by reducing its membrane permeability, preventing them from entering the cell [185]. When the membrane protein composition changes, the pores of the membrane transport system are altered, and, hence, antibiotics cannot pass through. Modifying the lipid A moiety of a lipopolysaccharide structure lowers the net negative charge of a membrane, decreasing its attraction for polymyxin and resistance to this antibiotic [186].

*Salmonella* can also pump out a drug before it reaches the target site, using efflux pumps or multidrug resistance pumps. Many antibiotics, such as ß-lactams, fluoroquinolones, and carbapenems, can be pumped out using this mechanism [182]. The genes encoding these efflux pumps (e.g., *qepA* and *oqxAB*) are positioned in mobile elements, such as plasmids [187]. *Salmonella* can also exert resistance by using chromosomal-linked gene products through mutations that code for the drug’s target or the mechanisms that control the internalization of the drugs [185]. Single point mutations in chromosomal *parC* and *parE*, topoisomerase genes responsible for quinolone resistance, and the DNA gyrase genes *gyrA* and *gyrB* have been reported in some *Salmonella* serotypes. The resulting alterations cause the bacterium to be resistant to fluoroquinolones and quinolones [188].

### 7.2. Spread of Antimicrobial Resistance

The indiscriminate use of antimicrobial agents other than therapeutic agents, such as the case of growth promoters in food animals and the sharing of human drugs for treating animal infections or other veterinary use, is considered the leading cause of the emergence of resistance of microbes to the first-line as well as the last resort antimicrobial agents [158,173]. MDR *Salmonella* strains have been transmitted from animals to humans by consuming water or food contaminated with the animals’ feces, eating contaminated food from animal sources, or having direct contact with infected subjects [189]. Furthermore, MDR *Salmonella* strains have been discovered in several exotic pet animals, such as turtles and tortoises, and in their aquatic habitat, potentially exposing individuals to contagious zoonotic infections [190].

### 7.3. Clinical Importance

The emergence of MDR *Salmonella* serotypes which have developed resistance to multiple antimicrobials substantially influences the treatment of human salmonellosis with antibiotics. Invasive serotype infections are typically fatal and require prompt antibiotic therapy. Third-generation cephalosporins and quinolones have long been the treatment of choice for MDR *Salmonella* infections [191]. The *Salmonella* serotypes showing resistance to quinolones and cephalosporins present a new problem in treating infected individuals. The lack of successful antibiotic therapy may result in higher morbidity and fatality [192]. As a result of the introduction of MDR *Salmonella*, the severity of bacterial infections in people and animals has risen. According to epidemiological research, MDR *Salmonella* strains generate more severe or fatal infections than non-MDR strains [193,194].

## 8. Prevention of Salmonellosis

Enteric fever spreads chiefly through the fecal–oral route. Historically, enteric fever was prevalent in the U.S. and Western Europe; however, typhoid-causing *Salmonella* infection has dramatically reduced with appropriate water and food sanitation, hygienic practices, pasteurization of dairy products, and the prohibition of using human feces as a fertilizer. In Latin America, the occurrence of *Salmonella* infections has been reduced with improved sanitary measures. Availability of safe food and drinking water, good sanitation, and the utilization of typhoid vaccines are currently the mainstays of enteric fever prevention [195].

The primary objective for eliminating the potential transmission pathways of typhoid *Salmonella* and NTS is to ensure the safety of drinking water. This critical step has been implemented effectively in developed nations, such as the U.S. and in Europe, but not in underdeveloped and developing continents [196]. Apart from water, *Salmonella* spp. may be found in several foods, including chicken, eggs, meats, nuts, flour, vegetables, and dairy products. Proper food handling and preparation are recommended to eliminate bacterial contamination of food. Food irradiation has been widely advocated in many nations because of its efficiency in lowering the danger of food contamination. Food irradiation technology has been accepted by various public health bodies, including the WHO and the CDC [197,198].

Vaccination is one of the crucial strategies to prevent enteric fever infections [199,200]. The two vaccinations now licensed to avoid EF are inactive oral and parenteral live attenuated vaccines [201]. These approved vaccines, however, are only for newborns, and are ineffective in preventing NTS and *S.* Paratyphi infections [202]. One promising and efficient solution to manage NTS is to limit antimicrobials, especially human-use antibiotics, in the feed of food animals and live animals [173,194,203]. In addition, plant-derived antimicrobials, probiotics, or direct-fed-microbes (DFM) are applied to control *Salmonella* in meat-producing animals and poultry [204,205,206,207,208].

## 9. Future Perspective and Conclusions

Human salmonellosis is still a significant public health threat across the world and an important cause of foodborne outbreaks all over the globe. This review emphasized providing data on recent episodes of salmonellosis reported in different clinical settings and geographical regions, including Africa, the Middle East and North Africa, Europe, and the USA in the farm-to-fork continuum. *Salmonella* strains’ genetic makeup allows them to withstand various situations, including animal, human, and non-animal hosts. The organism possesses a wide range of virulence factors involved in the multiple stages of infection. Their cunning survival strategy makes it challenging to eliminate this bacterium from the farm-to-fork food supply chain continuum and makes the infection cycles more difficult to control.

Furthermore, the emergence of MDR *Salmonella* strains has led to a challenging situation in treating the disease caused by these strains. As we discussed, several *Salmonella* outbreaks have been reported in different continents associated with various foodstuffs. However, the mortality and morbidity rate of the disease varied, reflecting differences in prevalence in food and animals used for food production, animal trade between countries, the proportion of travel-associated cases, and the quality and coverage of surveillance systems. Several preventative strategies have been recommended to reduce the spread of *Salmonella* infection, including restricting indiscriminate antibiotic use in food animals, which is by far the most successful. In addition, plant-based antimicrobials, probiotics, and bacteriophages are considered viable alternatives as feed additives. For the prevention of enteric fever, two vaccines have been approved; however, there are no certified vaccinations for *S.* Paratyphi and NTS infections. More studies into the preparation of vaccines for all *Salmonella* strains are needed.

The role of ecology in transmitting *Salmonella* from a One Health (a multidisciplinary and cross-sectoral cooperation) perspective is a crucial approach to managing the disease in the farm-to-fork continuum. Hence, emphasis should be given to the safe and efficient control of insects and rodents, which are the main vectors of contamination and cross-species transmission. In addition, in a free-range environment, safe and efficient barriers are required to prevent the transmission of pathogens among cattle, chickens, and pigs through contaminated feed (e.g., pasture) or the environment.

The mechanisms of *Salmonella* persistence outside the host are an essential part of their lifecycle and a prerequisite for their evolutionary success as human pathogens. Therefore, detailed investigations are needed to examine the transmission of diseases mainly associated with low-moisture foods, such as peanut butter, spray-dried milk, dry cereal, infant formula, nuts, etc. Furthermore, from a public health point of view, thorough investigations are needed to understand the bacterial factors or other related factors involved in the persistence of the pathogen in chronic infections and the vertical transmission of the agent. 

Increasing awareness and intensive implementation of food safety pillars could effectively combat foodborne diseases, including human salmonellosis. All stakeholders, including consumers, farmers, food vendors, and others involved in the food system, must be formally and informally educated on the basic steps of food safety. Food safety training can be conducted in the workplace, school, or community space, via video talks/demonstrations, on any social media platforms (Facebook, Twitter, Instagram, etc.), talk shows, radio interviews, or media briefings. Furthermore, community activities can be initiated by organizing a walk, run, bicycle ride, or fitness classes to publicize food safety as a key to sustaining life and promoting good health.

## Figures and Tables

**Table 1:** Summary of worldwide *Salmonella* causing diarrheal diseases (from 2019–2022).

Year	*Salmonella enterica* Serovar	No of Cases	Source of Country	Food Source (s)	References
2018	Concord	NA	Israel	Tahini products	[3]
2018	Unidentified serovar	NA	Australia	Chicken sandwich	[3]
2019	Unidentified serovar	NA	USA	Backyard poultry	[3]
2021	Oranienburg	1040	USA	Onion	[129]
2022	Typhimurium	324	Europe and USA	Chocolate products	[162]
2022	Enteritidis	NA	Canada	Exposure to live mice	[163]
2017 and 2019	Multiple serovars	325	United States	Whole, fresh Maradol papayas	[164]
2019	Heidelberg	164 (48.5%)	North West Province, South Africa	School lunch at public primary day school	[165]
2019	Newport	25	Sweden	Imported frozen cooked crayfish in dill brine	[166]
2019	Oranienburg	26	USA (14 states)	Contact with pet turtles	[167]
2019	Six different serovars: Amsterdam, Havana, Kintambo, Mbandaka, Orion, and Senftenberg)	121	Five EU/EEA countries	Imported sesame-based products (originating from Syria)	[133]

NA—not available.

**Table 2:** Complete susceptibility and multi-drug resistance (MDR) in *Salmonella* spp. isolated from humans in 2020 in Europe.

Country *	Susceptibility to All Tested Antibiotics (%)	Multi-Resistant (%)
Austria (N = 894)	61.5	12.9
Belgium (N = 706)	39.8	35.0
Denmark (N = 252)	71.8	19.8
Estonia (N = 95)	68.4	22.1
France (N = 713)	64.7	19.9
Ireland (N = 713)	65.4	12.6
Italy (N = 872)	42.7	42.9
Luxembourg (N = 90)	41.1	31.1
Netherlands (N = 494)	55.3	28.9
Portugal (N = 238)	50.8	29.8
Romania (N = 36)	38.9	11.1
Slovenia (N = 172)	62.8	19.8
Spain (N = 766)	55.6	24.2
Sweden (N = 344)	77.6	15.1
Total	55.9	25.4

* Tested antibiotics: gentamicin, ampicillin, chloramphenicol, cefotaxime, ceftazidime, tigecycline, meropenem, nalidixic acid ciprofloxacin, colistin, azithromycin, sulfamethoxazole, co-trimoxazole, trimethoprim, and tetracycline. Source: [174].

## Data Availability

Not applicable.
